# Hepatic tumor classification using texture and topology analysis of non-contrast-enhanced three-dimensional T1-weighted MR images with a radiomics approach

**DOI:** 10.1038/s41598-019-45283-z

**Published:** 2019-06-19

**Authors:** Asuka Oyama, Yasuaki Hiraoka, Ippei Obayashi, Yusuke Saikawa, Shigeru Furui, Kenshiro Shiraishi, Shinobu Kumagai, Tatsuya Hayashi, Jun’ichi Kotoku

**Affiliations:** 1 Graduate School of Medical Care and Technology, Teikyo University, 2-11-1 Kaga, Itabashi-ku, Tokyo 173-8605 Japan; 2 Institute for the Advanced Study of Human Biology (ASHBi), Center for Advanced Study, Kyoto University Institute for Advanced Study (KUIAS), Kyoto University, Yoshida, Ushinomiya-cho, Sakyo-ku, Kyoto 606-8501 Japan; 3 Center for Advanced Intelligence Project, RIKEN, 1-4-1 Nihonbashi, Chuo-ku, Tokyo 103-0027 Japan; 4 Department of Radiology, Teikyo University School of Medicine, 2-11-1 Kaga,, Itabashi-ku, Tokyo 173-8605 Japan; 5 Central Radiology Division, Teikyo University Hospital, 2-11-1 Kaga, Itabashi-ku, Tokyo 173-8606 Japan

**Keywords:** Machine learning, Cancer imaging, Applied mathematics

## Abstract

The purpose of this study is to evaluate the accuracy for classification of hepatic tumors by characterization of T1-weighted magnetic resonance (MR) images using two radiomics approaches with machine learning models: texture analysis and topological data analysis using persistent homology. This study assessed non-contrast-enhanced fat-suppressed three-dimensional (3D) T1-weighted images of 150 hepatic tumors. The lesions included 50 hepatocellular carcinomas (HCCs), 50 metastatic tumors (MTs), and 50 hepatic hemangiomas (HHs) found respectively in 37, 23, and 33 patients. For classification, texture features were calculated, and also persistence images of three types (degree 0, degree 1 and degree 2) were obtained for each lesion from the 3D MR imaging data. We used three classification models. In the classification of HCC and MT (resp. HCC and HH, HH and MT), we obtained accuracy of 92% (resp. 90%, 73%) by texture analysis, and the highest accuracy of 85% (resp. 84%, 74%) when degree 1 (resp. degree 1, degree 2) persistence images were used. Our methods using texture analysis or topological data analysis allow for classification of the three hepatic tumors with considerable accuracy, and thus might be useful when applied for computer-aided diagnosis with MR images.

## Introduction

Magnetic resonance (MR) imaging is an important tool for detection and differential diagnosis of hepatic tumors. The differential diagnosis of hepatic tumors is performed by comparative observation of MR images obtained by multiple sequences (e.g., T1-weighted images, T2-weighted images, T1-weighted chemical shift images, and diffusion-weighted images)^1–8^. For differential diagnosis, dynamic T1-weight contrast-enhanced imaging using extracellular fluid gadolinium-based contrast agents (e.g., gadopentetic acid, gadodiamide, gadoteric acid, gadoteridol and gadobutrol) or a hepatocyte-specific gadolinium-based contrast agent (gadoxetate disodium [Gd-EOB-DTPA]) is helpful^1–10^, although the use of those agents is contraindicated in patients with renal insufficiency^11,12^. Even so, a differential diagnosis cannot usually be made from non-contrast-enhanced T1-weighted MR images alone because most hepatic tumors appear as low signal intensity areas in the liver without pathognomonic findings^1–8^. From a different perspective, three-dimensional (3D) MR images of the liver consist of gray-scale values of numerous voxels. Nevertheless, 3D arrays of the gray-scale values in hepatic tumors might present specific distinctive geometric patterns to tumor types, even if they are unrecognizable visually.

The development of radiomics, an emerging field in medicine, has been facilitated by progress in high-throughput computing. It is designed to extract multiple quantitative features (radiomics features) from radiologic images (e.g., computed tomography [CT], MR imaging, and positron emission tomography) and to analyze them for various specific medical purposes (e.g., decoding tissue pathology and producing a prognosis or therapeutic response for some pathologic condition)^13–18^. In radiomics, texture features, which describe statistical relations between voxels with distinctive contrast values, have often been used as primary quantitative features^13–18^. Topological data analysis is a recently advanced concept in applied mathematics aimed at characterization of shapes in complex data. Persistent homology is the main tool used for topological data analysis and has been applied in various scientific fields (e.g., materials science, chemistry, engineering, astronomy, biology and medicine)^19–30^. Persistent homology provides a multiscale description of topological features (e.g., connected components, rings, and cavities) in a dataset, and a persistence diagram, a visualization of persistent homology as a two-dimensional (2D) histogram, has been used as the principal descriptor for data analysis. This article describes our first experience with hepatic tumor classification by characterization of non-contrast-enhanced 3D T1-weighted MR images using texture features and persistent homology and using analysis of the obtained data with machine learning models.

## Results

The maximum diameters of the lesions on axial MR images were 0.7–10.2 cm (mean ± standard deviation [SD], 2.9 ± 2.1 cm; median, 2.1 cm; interquartile range [IQR], 1.4–4.0 cm), 0.7–5.8 cm (mean ± SD, 2.2 ± 1.1 cm; median, 2.0 cm; IQR, 1.4–2.6 cm), and 0.6–4.6 cm (mean ± SD, 1.8 ± 1.0 cm; median, 1.4 cm; IQR, 1.0–2.4 cm), respectively, for HCC, MT, and HH. The numbers of voxels in the rectangular parallelepiped ROIs, each containing a tumor, were 4,032–223,975 (mean ± SD, 23,853 ± 37,869; median, 10,134; IQR, 6,980–20,172), 1,350–34,425 (mean ± SD, 6,560 ± 5,651; median, 5,143.5; IQR, 3,896–6,600), and 1,176–26,240 (mean ± SD, 5,941 ± 5,385; median, 3,832.5; IQR, 2,977–6,250), respectively, for HCC, MT, and HH.

Table 1 presents results of our classification obtained through texture analysis. For the classification of HCC and MT (resp. HCC and HH, MT and HH), we obtained accuracy of 92% (resp. 90%, 73%) with the sensitivity, specificity, and area under the curve (AUC) respectively being 100% (resp. 96%, 72%), 84% (resp. 84%, 74%), and 0.95 (resp. 0.95, 0.75).

**Table 1:** Classification results for hepatocellular carcinoma (HCC), metastatic tumor (MT) and hepatic hemangioma (HH) using texture features and linear discriminant analysis (LDA) with an elastic net penalty.

Subjects	Accuracy	Sensitivity	Specificity	AUC^#^
HCC and MT	0.92 (92/100)	1.00 (50/50)	0.84 (42/50)	0.95
HCC and HH	0.90 (90/100)	0.96 (48/50)	0.84 (42/50)	0.95
MT and HH	0.73 (73/100)	0.72 (36/50)	0.74 (37/50)	0.75

^#^AUC = area under the curve.

Table 2 shows results of our classification with topological data analysis. In the classification of HCC and MT (resp. HCC and HH), we obtained the best accuracy of 85% (resp. 84%) when feature vectors obtained from degree 1 persistence images and XGBoost were used, with the sensitivity, specificity, and AUC, respectively being 86% (resp. 86%), 84% (resp. 82%) and 0.85 (resp. 0.89). In the classification of MT and HH, we obtained the best accuracy of 74% when the feature vectors obtained from degree 2 persistence images and XGBoost were used, with the sensitivity, specificity, and AUC, respectively being 68%, 80%, and 0.71.

**Table 2:** Classification results for hepatocellular carcinoma (HCC), metastatic tumor (MT) and hepatic hemangioma (HH) from persistence images of three types (degree 0, degree 1, and degree 2) using logistic classifier with an elastic net penalty and XGBoost.

Subjects	MLM*	Degree	Accuracy	Sensitivity	Specificity	AUC^#^
HCC and MT	Logistic	0	0.69 (69/100)	0.48 (24/50)	0.90 (45/50)	0.69
		1	0.75 (75/100)	0.66 (33/50)	0.84 (42/50)	0.78
		2	0.70 (70/100)	0.58 (29/50)	0.82 (41/50)	0.69
	XGBoost	0	0.82 (82/100)	0.82 (41/50)	0.82 (41/50)	0.85
		1	0.85 (85/100)	0.86 (43/50)	0.84 (42/50)	0.85
		2	0.68 (68/100)	0.80 (40/50)	0.56 (28/50)	0.68
HCC and HH	Logistic	0	0.74 (74/100)	0.60 (30/50)	0.88 (44/50)	0.73
		1	0.73 (73/100)	0.56 (28/50)	0.90 (45/50)	0.73
		2	0.69 (69/100)	0.56 (28/50)	0.82 (41/50)	0.69
	XGBoost	0	0.79 (79/100)	0.66 (33/50)	0.92 (42/50)	0.83
		1	0.84 (84/100)	0.86 (43/50)	0.82 (41/50)	0.89
		2	0.72 (72/100)	0.64 (32/50)	0.80 (40/50)	0.71
MT and HH	Logistic	0	0.62 (62/100)	0.64 (32/50)	0.60 (30/50)	0.61
		1	0.56 (56/100)	0.18 (9/50)	0.94 (47/50)	0.49
		2	0.52 (52/100)	0.28 (14/50)	0.76 (38/50)	0.45
	XGBoost	0	0.64 (64/100)	0.74 (37/50)	0.54 (27/50)	0.60
		1	0.60 (60/100)	0.90 (45/50)	0.30 (15/50)	0.57
		2	0.74 (74/100)	0.68 (34/50)	0.80 (40/50)	0.71

*MLM = machine learning models.

^#^AUC = area under the curve.

**Figure 1:**
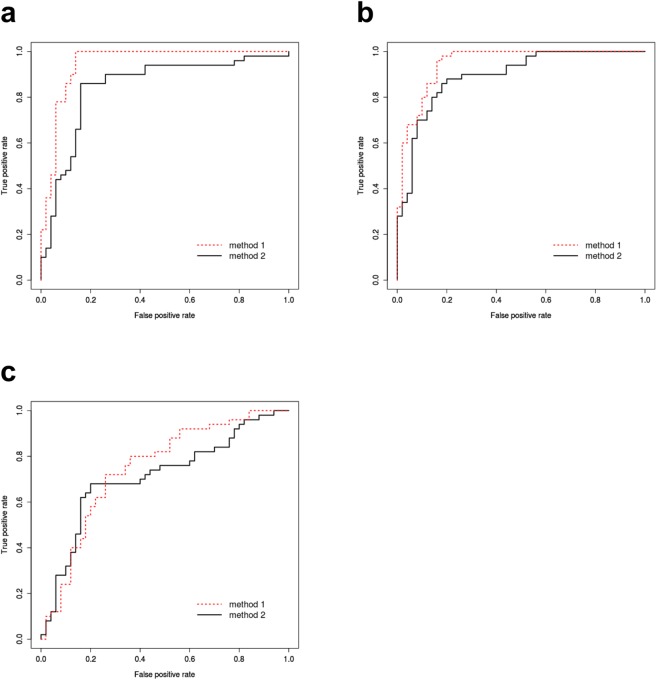
ROC curves obtained from texture analysis (method 1, dotted line) and topological data analysis using XGBoost (method 2, solid line). (**a**) Classification between HCC and MT. Method 2 uses degree 1 persistence images. The areas under the curve (AUC) are 0.95 and 0.85, respectively, for method 1 and method 2. (**b**) Classification between HCC and HH. Method 2 uses degree 1 persistence images. The AUCs are 0.95, and 0.89, respectively, for method 1 and method 2. (**c**) Classification between MT and HH. Method 2 uses degree 2 persistence images. The AUCs are 0.75 and 0.71, respectively, for method 1 and method 2.

Figure 1 portrays receiver operating characteristic (ROC) curves obtained from texture analysis and topological data analysis using XGBoost.

## Discussion

Inherent tissue contrast on MR images is determined primarily using three parameters: proton density, longitudinal relaxation time (T1), and transverse relaxation time (T2 or T2*)^31,32^. Other sources of tissue contrast include magnetization transfer, chemical shift, and flow. MR images visualize hepatic tumors based on the differences in these parameters between the tumor tissue and non-tumorous liver tissue. Such differences derive from the distinctive histologic features (e.g., types and density of cells, and interstitial structures including the vascular system and interstitial fluid) of these tissues, in which protons are contained. Associated pathologic conditions such as necrosis, interstitial fibrosis, fatty infiltration, calcification, and deposition of metals (e.g., iron) can also alter these parameters and thereby affect the signal intensity of the lesions and liver parenchyma^3,5–8,33–35^.

We used 3D T1-weighted MR images for the analyses. In these images, T1 contrast is accentuated reliably, but tissue contrast is also affected to a considerable extent by the proton density and transverse relaxation time^36,37^. On observation with the naked eye, non-contrast-enhanced T1-weighted MR images are generally not useful for the differential diagnosis of hepatic tumors because most of them appear as low signal intensity areas in the liver without pathognomonic findings. On the other hand, the MR images consist of numerous voxels lying in the lesions and surrounding liver parenchyma. Each voxel shows a gray-scale value according to its signal intensity, affected by the various histopathologic factors described above. For this reason, 3D arrays of gray-scale values in the lesions might present specific geometric patterns that are distinctive to tumor types, although they might be unrecognizable visually. Texture features and persistence images have the potential to capture such arrays of gray-scale values and might provide useful information for the differential diagnosis of hepatic tumors. Based on the possibility presented above, we attempted a classification of HCC (the most common primary liver malignancy), MT of the liver (the most common liver malignancy), and HH (the most common benign liver tumor) by characterization of 3D T1-weighted MR images using texture features and vectorized features of persistence images. In our classification method, both features were analyzed using machine learning models. Our method differentiated between HCC, MT, and HH with considerable accuracy. These results suggest the correctness of our approach and also the possible clinical usefulness of such methods when used in the computer-aided diagnosis of hepatic tumors with MR imaging.

Recently, deep learning with convolutional neural networks (CNNs) has been attracting attention as a promising tool for image classification^38,39^, even though CNNs require a massive amount of datasets. Our approaches by analyzing texture features or vectorized features of persistence images with machine learning models allowed for the classification of hepatic tumors despite the small number of datasets acquired from only 150 lesions, which would be insufficient for standard CNNs. These results indicate the benefit of both features in machine learning image classification.

Actually, HCC occurs mostly in patients with chronic liver diseases that cause liver fibrosis^1,3,33^. Consistent with this, chronic liver diseases were proven histologically in all but 1 of the 37 patients with HCC in our series too. In contrast, the incidence of chronic liver diseases is low in patients with MT or HH. In our results obtained using texture analysis and topological data analysis, the classification accuracies of HCC and MT, and HCC and HH tended to be higher than those of MT and HH. The presence of liver fibrosis or regenerative liver nodules in the HCC patients might have contributed to this tendency in our results as the MR images used for the analysis included the liver parenchyma at the peripheries.

We analyzed 3D T1-weighted MR images of the whole liver, taken as pre-contrast images of dynamic contrast-enhanced MR imaging, obtained in breath-hold with acquisition time of approximately 20 s. The spatial resolution of these images is limited because dynamic MR imaging requires adequate temporal resolution, presenting an unavoidable tradeoff between spatial and temporal resolutions^40,41^. Spatial resolution of our MR images can be improved by voxel size reduction achieved by decreasing the slice thickness^42^. Although such imaging covering the whole liver prolongs the acquisition time, the time can be shortened to an acceptable level by reducing the coverage area to only that part of the liver which contains the tumors. The use of such images with higher spatial resolution might improve the classification accuracy of our method because they offer more detailed gray-scale information that better reflects the histopathologic features of hepatic tumors.

We limited our analysis of 3D MR imaging data to only T1-weighted MR images because of the following reason. In our institution, T1-weighted, T2-weighted, T1-weighted chemical shift, diffusion-weighted and T1-weighted dynamic contrast-enhanced sequences are performed in routine MR imaging of hepatic tumors. Of these, 3D imaging data of the whole liver with breath-hold are obtained only in the dynamic contrast-enhanced sequence, while the other sequences provide 2D images during free breathing with respiratory gating. Thus we chose to use the pre-contrast images of the dynamic contrast-enhanced study and evaluated 3D arrays of the gray-scale values in hepatic tumors on the images using texture analysis and topological data analysis. Currently, 3D imaging of the liver with breath-hold is not generally performed for a T2-weighted or diffusion-weighted sequence, as its acquisition time is too long to be used clinically. When 3D data acquisition of the liver becomes available for such sequences hereafter, the data could be evaluated with similar methods to ours.

This study presents several limitations. First, only a few lesions could be evaluated. Second, the lesion size varied considerably with the maximum diameters on axial MR images of 0.7–10.2 cm. This variance likely influenced our results because the feature amounts of texture features and persistence images decrease in parallel with decreasing tumor volume. The relation between tumor size and classification accuracy must be addressed in future studies. Third, the 3D MR imaging data analyzed in this study were obtained with two MR systems of 1.5 T and 3.0T. Use of the data obtained with a single MR system might have increased the classification accuracy of our methods, as it would have improved the data integrity. Fourth, we used the data of 3D ROIs containing non-tumorous liver at the peripheries. Although the tumors occupied the majority of voxels in the ROIs, the coexistence of such non-tumorous parts might have affected the classification accuracy of our methods. Fifth, in this study, we used four selected features (i.e., GLCM, GLRLM, GLSZM and NGTDM) for the texture analysis in accordance with some previous reports of radiomics in oncology^16,17^. Additional use of other radiomics features (e.g., GLDM and shapes features) might also improve the classification accuracy. Last, although our method successfully classified MT from colorectal cancers and HCC or HH with considerable accuracy, this result cannot be applied to MTs from other primary malignancies as the histopathologic features of MTs differ essentially from one another since they resemble those of their primary malignancies^5,6^.

In conclusion, our methods using texture analysis or topological data analysis support the classification of HCC, MT, and HH with considerable accuracy, solely based on non-contrast-enhanced 3D T1-weighted MR images. These methods might be useful when used for the computer-aided diagnosis of hepatic tumors with MR imaging.

## Methods

### Study population and image acquisition

Non-contrast enhanced fat-suppressed 3D T1-weighted gradient echo MR images of 150 hepatic tumors obtained in our institution were used for our analysis. These images were taken as pre-contrast images of dynamic contrast-enhanced MR imaging of the liver using an extracellular fluid gadolinium-based contrast agent or hepatocyte-specific gadolinium-based contrast agent. The lesions comprised 50 hepatocellular carcinomas (HCCs), 50 metastatic tumors (MTs) from colorectal cancers and 50 hepatic hemangiomas (HHs) found in a total of 93 patients. The HCC patient group comprised 37 consecutive patients (30 males and 7 females, aged 55 to 85 years) who had undergone dynamic contrast-enhanced MR imaging of the liver followed by histological examination of the tumors from November 2009 to April 2017. In them, the histological diagnosis of HCC was made using tumor specimens obtained by surgical resection (n = 33) or percutaneous needle biopsy (n = 4) of one or more lesions. Histological examination of the liver parenchyma simultaneously performed showed the presence of chronic liver disease (liver chirrosis, n = 19; chronic hepatitis, n = 11; liver fibrosis, n = 6) in all but one of the 37 patients. The MT patient group comprised 23 consecutive patients (17 males and 6 females, aged 43 to 79 years) who had undergone dynamic contrast-enhanced MR imaging from December 2010 to September 2017. In them, the diagnosis of MT was made clinically based on a personal history of colorectal cancer (rectal cancer, n = 15; sigmoid colon cancer, n = 6; descending colon cancer, n = 2) and the radiological features of the hepatic lesions on dynamic contrast-enhanced CT and MR imaging. For 26 of the 50 MTs, the diagnosis was histologically confirmed with surgical or biopsy specimens. The HH patient group comprised 33 consecutive patients (13 males and 20 females, aged 31 to 81 years) who had undergone dynamic contrast-enhanced MR imaging from May 2015 to September 2017. In them, the diagnosis of HH was made radiologically based on markedly high signal intensity of the lesions on T2-weighted MR images along with their characteristic contrast enhancement pattern on dynamic contrast-enhanced CT and MR imaging^7,8^.

MR imaging was performed using 1.5T (Avanto; Siemens Healthineers, Erlangen, Germany) or 3.0T (Skyra; Siemens Healthineers, Erlangen, Germany) MR systems. Of the 150 hepatic tumors described above, 122 (46 HCCs, 43 MTs and 33 HHs) were examined with the 1.5 T system, and the other 28 (4 HCCs, 7 MTs and 17 HHs) with the 3.0T system. Non-contrast-enhanced fat-suppressed 3D T1-weighted gradient echo MR images were obtained in the axial plane using a phased-array multicoil for the body during breath-holding: 3.20–5.26 ms time of repetition (TR); 1.13–1.50 ms time of echo (TE); 15° and 10° flip angles, respectively, for 1.5 T and 3.0T systems; number of excitations (NEX), 1; 3.0 or 3.5 mm slice thickness; no intersection gap; and 15–24 s acquisition time. The fields-of-view (FOV) ranged from 30.0–40.0, 22.5–30.0, and 16.8–24.0 cm, respectively, in the x-, y-, and z-directions. The matrix sizes were 256–288 and 192–216, respectively, in the x and y directions. In this way, the voxel size was 1.04–1.56 mm and was of the same length in the x- and y-directions and 3.0 or 3.5 mm in the z-direction.

### Preparation of input data

Input lesion images were prepared in the following steps: Digital Imaging and Communication in Medicine (DICOM) data of the MR image of the liver were anonymized using in-house software. For each of the 150 tumors, a 3D region of interest (ROI) in the shape of a rectangular solid, enclosing the whole lesion, was created by a diagnostic radiologist with 20 years of experience. The ROIs contain the liver parenchyma surrounding the lesions at the peripheries. Some of them also contain fat tissue around the liver at the peripheries. Image data of the 150 ROIs consisting of signal intensities of the voxels were extracted.

### Texture analysis

#### Extraction of texture features

To verify the classification accuracies of the three hepatic tumors using texture analysis, we extracted texture features of the 150 ROIs. Texture features have been used to quantify tumor characteristics in radiomics analyses^13–18^. For this study, we extracted global features based on the first-order histogram and matrix-based features, and obtained 43 texture features from a lesion in accordance with a previously reported model^16,17^. Table 3 presents the texture features used for this study.

**Table 3:** Texture features and texture extraction parameters used for this study.

Texture features		
Feature index	Texture type	Texture name
1–3	Global	Variance, Skewness, Kurtosis
4–12	GLCM	Energy, Contrast, Correlation, Homogeneity, Variance, Sum Average, Entropy, Dissimilarity, Auto Correlation
13–25	GLRLM	Short Run Emphasis, Long Run Emphasis, Gray-Level (GL) Non-uniformity, Run-Length Non-uniformity, Run Percentage, Low GL Run Emphasis, High GL Run Emphasis, Short Run Low GL Emphasis, Short Run High GL Emphasis, Long Run Low GL Emphasis, Long Run High GL Emphasis, GL Variance, Run-Length Variance
26–38	GLSZM	Small Zone Emphasis, Large Zone Emphasis, GL Level Non-uniformity, Zone-Size Non-uniformity, Zone Percentage, Low GL Zone Emphasis, High GL Zone Emphasis, Small Zone Low GL Emphasis, Small Zone High GL Emphasis, Large Zone Low GL Emphasis, Large Zone High GL Emphasis, GL Variance, Zone-Size Variance
39–43	NGTDM	Coarseness, Contrast, Busyness, Complexity, Strength
**Texture extraction parameters**		
Wavelet band-pass filtering	1/2, 2/3, 1, 3/2, 2	
Isotropic voxel size at resampling	initial in-plane resolution, 1, 2, 3, 4, 5	
Number of gray levels at quantization	8, 16, 32, 64	
Quantization algorithm	‘Equal-probability’, ‘Lloyd–Max’	

GLCM: Gray-level co-occurrence matrix.

GLRLM: Gray-level run-length matrix.

GLSZM: Gray-level size zone matrix.

NGTDM: Neighborhood gray-tone difference matrix.

#### Selection of texture extraction parameters

Image processing by texture extraction parameters makes texture features more expressive of tumor characteristics. Here, the texture extraction parameter denotes the combination of the following parameters: the ratio related with wavelet band-pass filtering, isotropic voxel size at resampling, some gray levels, and quantization algorithm (Table 3). The number of extraction parameters for each feature becomes 240 as the number of all combinations of texture extraction parameters.

Before classification, these texture extraction parameters were uniquely selected for each feature to suppress the decrease in the stability of the classification model by multicollinearity. The features with each texture extraction parameter were compared with the binary outcome variables labeling the lesion, and the parameters with the highest correlation with the outcome variables were selected for each feature. For this study, we used Spearman’s rank-order correlation to calculate the correlations between features and outcome variables. Extracted texture features with the parameters with the highest correlation become more expressive of tumor characteristics. Furthermore, use of the bootstrap method in the texture selection step enhances the liability of correlation calculation^43^. After the selection of texture extraction parameters, 43 features with optimal parameters were extracted.

The texture features extraction and parameters selection steps were performed using the Matlab Image Processing Toolbox (version 9.2), Signal Processing Toolbox (version 10.0), Statistics and Machine Learning Toolbox (version 11.1), and Wavelet Toolbox (version 4.18).

#### Statistical analysis

Classification of hepatic tumors with texture features was performed using linear discriminant analysis (LDA) with an elastic net penalty. The model parameters were determined using a grid search. We used these classifiers and leave-one-out cross-validation (LOOCV), a model evaluation method, to verify the classification accuracy of our method. The dataset was separated into one test datum and an *N* – 1 training set, where *N* stands for the number of data. The classifier model was learned by the training set. Then test data were predicted using the learned model. The classification step was conducted using the Matlab Statistics and Machine Learning Toolbox (version 11.1).

### Topological data analysis

#### Persistent homology and persistence diagram

**Figure 2:**
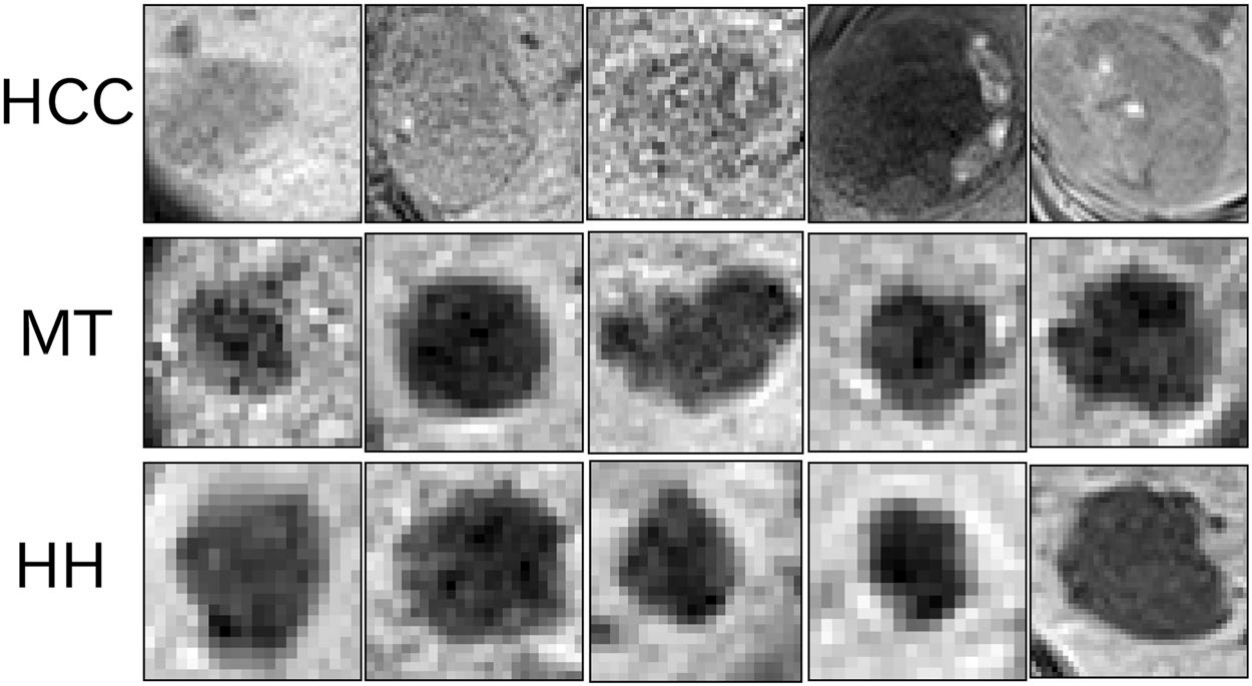
Gray-scale images of rectangular parallelepiped ROIs: axial views around the mid-section: five images each of the ROIs that contain HCC (top), MT (middle), and HH (bottom). We generated persistence diagrams of the ROIs from these voxel values.

This section introduces new radiomics features based on persistent homology. Before starting this topological data analysis, gray-scale values of the voxels in each ROI were normalized continuously from 0 to 255. Figure 2 shows axial gray-scale images of the ROIs created from the normalized values.

**Figure 3:**
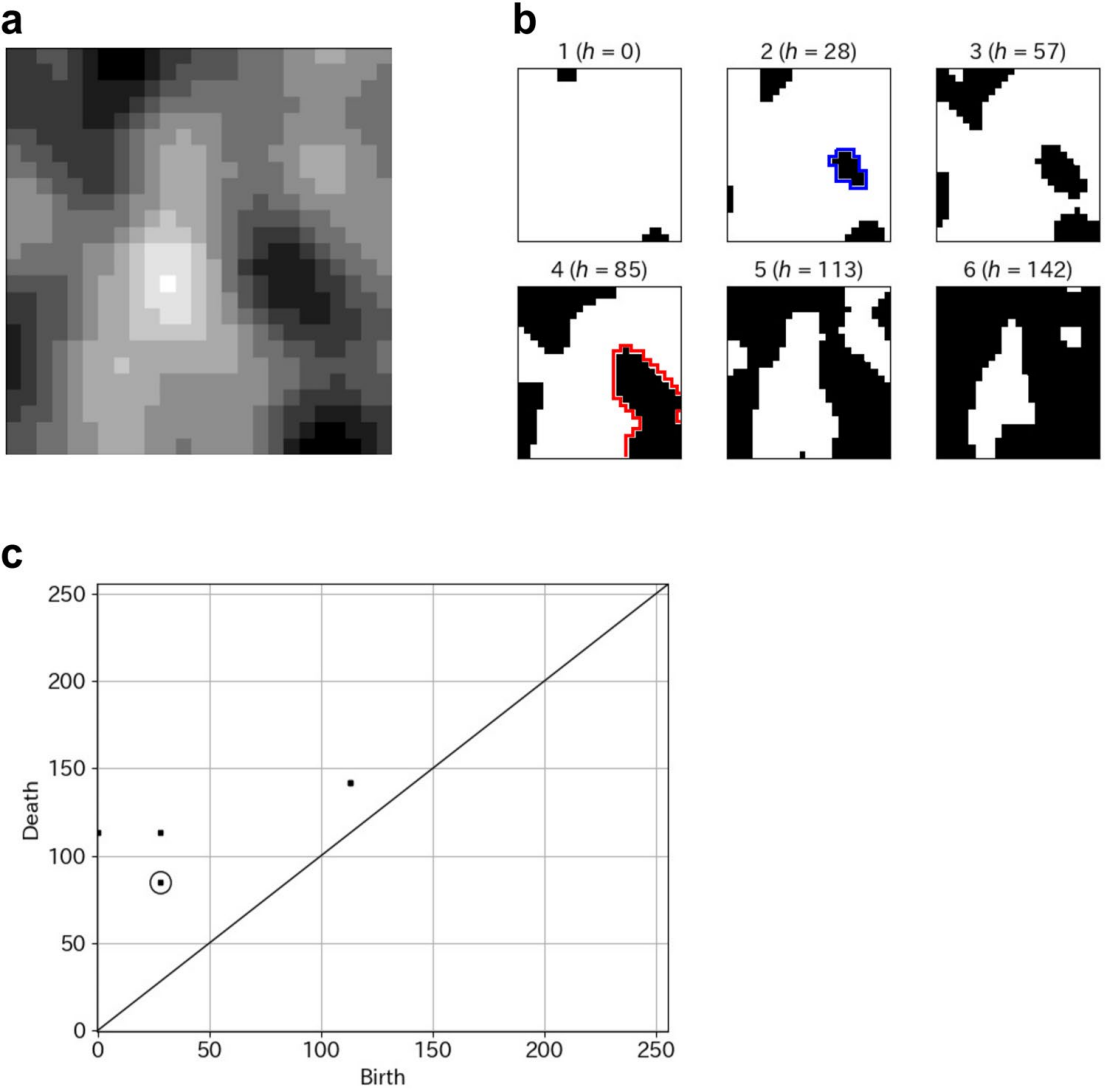
(**a**) A gray-scale image. (**b**) Filtration of binary images (*h*, threshold gray-scale value). A sequence of binary images is obtained by changing the threshold. The area surrounded by the blue line represents an example of the birth of a connected component. The area surrounded by the red line represents the death of the connected component. (**c**) Persistence diagram (degree 0). The point in the circle corresponds to the birth–death described above a pair of the connected component.

Given a grayscale digital image *X*, persistent homology captures topological features embedded in *X*: e.g., connected components, rings, and cavities^19–22^. The persistence diagram is a compact expression of the persistent homology constructed in the following manner. For each threshold value *h* of the gray-scale, we binarize the original gray-scale image into *X*_*h*_ and obtain a sequence of those binary images by changing the threshold to 0 ≤ *h* ≤ 255. Figure 3a,b present a sample of a gray-scale image and filtration images. In this sequence of binary images, each connected component created by black voxels has two specific thresholds, i.e., *h* = *b* (resp. *h* = *d*) at which the connected component is generated (resp. is dead). Values (*b*, *d*) are called the birth–death pair of the connected component. The degree 0 persistence diagram is the collection of all birth–death pairs of connected components appearing in the binary image sequence. The degree 1 and degree 2 persistence diagrams are defined respectively in the same manner applied to the ring and cavity. One important fact is that a birth–death pair with smaller lifetime (the difference in birth value and death values) is less significant because the corresponding connected component (or, ring, cavity) becomes dead quickly after being generated. The persistence diagram is usually visualized as a histogram on the plane, where the *x*-axis (resp. *y*-axis) expresses the birth value (resp. death value). Figure 3c presents a persistence diagram of a sample image.

For this study, we generated persistence diagrams of three types (degree 0, degree 1 and degree 2) for the 150 ROIs each containing a hepatic tumor using HomCloud^22,25,44^, a topological data analysis software package written in Python. All persistence diagrams were square [–0.5, 255.5]^2^ images.

#### Persistence image

Persistence diagrams must be vectorized for application to machine learning models. For this reason, we obtained persistence images from persistence diagrams^22,23^.

**Figure 4:**
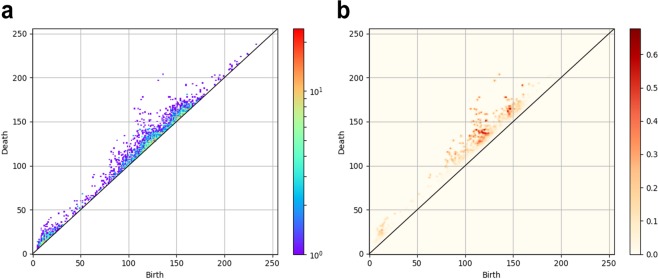
(**a**) Persistence diagram (degree 1) of an ROI containing HCC. Numerous dots denote birth–death pairs of cavities above the line of *y* = *x*. The dot colors reflect the number of birth–death pairs created at the points. (**b**) Persistence images were obtained from this persistence diagram. The dot color density is determined by the importance level in data characterization.

Given a degree *q* persistence diagram ${D}_{q}=\,\{({b}_{k},\,{d}_{k}):k=1,\,\ldots ,\,l\}$, where *l* denotes the number of birth–death pairs, persistence image *ρ* is defined by the weighted sum of Gaussian distributions on a plane as

$$\rho (x,\,y)=\sum _{k=1}^{l}w({b}_{k},\,{d}_{k})\exp (-\frac{{({b}_{k}-x)}^{2}+{({d}_{k}-y)}^{2}}{2{\sigma }^{2}})\,{\rm{and}}$$

$${w}({b},\,{d})=\arctan ({C}{({d}-{b})}^{{\boldsymbol{p}}})$$

here, *C* > 0, *ρ* > 0, σ > 0 are parameters; *w*(*b*, *d*) is a weight function. The weight function is chosen so that we can respect the significance of generators according to their lifetimes in statistical analysis. For this study, the persistence image parameters were found using a grid search. Figure 4 depicts a persistence diagram (degree 1) of an ROI including HCC and its persistence image.

**Figure 5:**
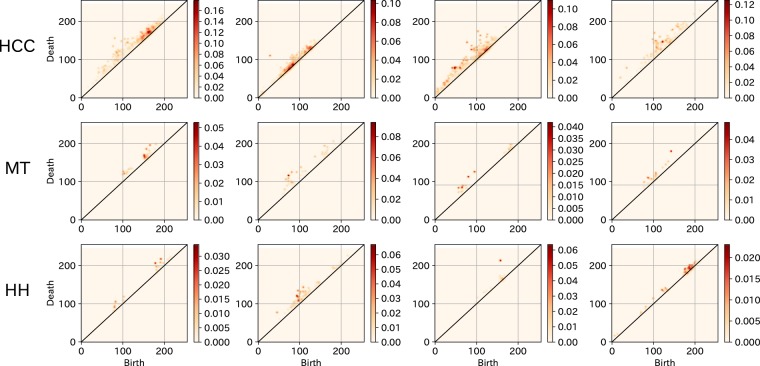
Persistence images of ROIs including tumors: four persistence images (degree 1) each of the ROIs that include HCC (top), MT (middle) and HH (bottom). The dot color density is determined by the importance level in data characterization.

For computation, we discretized persistence image *ρ* and constructed a histogram on the plane with an appropriate finite mesh. Then we obtained a vector from the discretization of *ρ* by ordering the elements on the grids in a prefixed order. The vector dimension is equal to the number of grids used for the histogram. The mesh for the discretized persistence images was obtained by dividing the square [–0.5, 255.5]^2^ into 256 × 256 grids. Figure 5 depicts some examples of persistence images (degree 1) of ROIs containing HCC, MT, and HH. All topological features extraction steps were conducted in Python (version 3.6.0; Python Software Foundation, Wilmington, Del).

#### Statistical analysis

To classify HCC, MT, and HH, feature vectors obtained from persistence images of three types (degree 0, degree 1 and degree 2) for the 150 ROIs were inputted into two machine learning models: a logistic classifier model with an elastic net penalty (glmnet package in R, ver. 2.0–10) and extreme gradient boosting (XGBoost) (xgboost package in R, ver. 0.6–4)^45^. We used these classifiers and LOOCV. For this study, the classifier model parameters were optimized based on a five-fold cross-validated grid search.

### Ethical statement

This study was approved by the Institutional Ethics Review Board (Teikyo University Review Board 17–108–2), and waived the need for written informed consent from patients, as long as patient data remained anonymous. All of the methods were carried out in accordance with the Declaration of Helsinki.

## Data Availability

The datasets analysed during the current study are available from the corresponding author on reasonable request.
